# Sinonasal Histopathological Changes After 12 Months of Dupilumab Treatment in Patients with Chronic Rhinosinusitis with Nasal Polyps

**DOI:** 10.3390/medsci14060686

**Published:** 2026-10-19

**Authors:** Bartłomiej Kamiński, Dominika Ochab, Janusz Kopczyński, Piotr Łacwik, Magdalena Bętkowska, Stanisław Flaga, Cezary Pałczyński

**Affiliations:** 1 Department of Otolaryngology, Maria Skłodowska-Curie District Hospital, 26-110 Skarżysko-Kamienna, Poland; 2 Collegium Medicum, Jan Kochanowski University, 25-369 Kielce, Poland; 3 Clinical Division of Lung Diseases and Allergology, Holy Cross Centre for Lung Disease, 26-060 Czerwona Góra, Poland; 4 Department of Oncological Pathology, Holy Cross Cancer Centre, 25-734 Kielce, Poland; 5 AGH University of Krakow, Faculty of Mechanical Engineering and Robotics, 30-059 Krakow, Poland

**Keywords:** chronic rhinosinusitis with nasal polyps, CRSwNP, dupilumab, tissue remodeling, eosinophils, Charcot–Leyden crystals

## Abstract

**Background/Objectives:** Dupilumab blocks the interleukin-4 receptor alpha (IL-4Rα) subunit, thereby inhibiting IL-4- and IL-13-mediated signaling, and is an effective treatment for severe chronic rhinosinusitis with nasal polyps (CRSwNP). This study assessed clinical response and histopathological changes reflecting eosinophilic inflammation and tissue remodeling after 12 months of dupilumab therapy. **Methods:** This prospective pilot real-world study included 27 patients with severe CRSwNP. Paired sinonasal mucosal biopsies were obtained before treatment and after 12 months. Histopathological assessment included tissue eosinophilia, basement membrane thickness, subepithelial edema, tissue neutrophilia, and Charcot–Leyden crystals (CLCs). Clinical outcomes included Nasal Polyp Score (NPS), Sino-Nasal Outcome Test-22 (SNOT-22), and the 12-item Sniffin’ Sticks test. **Results:** Median NPS decreased from 6.0 [5.5–7.0] to 1.0 [1.0–1.0] (*p* < 0.001), SNOT-22 from 73.1 ± 15.7 to 14.9 ± 10.3 (*p* < 0.001), and olfactory scores increased from 2.85 ± 2.46 to 7.54 ± 3.19 (*p* < 0.001). Tissue eosinophils decreased from 122 [81–323] to 36 [19.5–76] cells/mm^2^ (*p* < 0.001), basement membrane thickness from 6.0 [4.15–7.5] to 4.0 [3.15–5.0] µm (*p* = 0.0019), and subepithelial edema from grade 2 [2–3] to 1 [1–1] (*p* < 0.001). Tissue neutrophil counts did not change significantly, while CLC prevalence decreased from 7/27 (25.9%) to 0/27 (0%; *p* = 0.0156; Holm-adjusted *p* = 0.0469). **Conclusions:** Twelve months of dupilumab therapy was associated with marked clinical improvement and significant histopathological changes in the sinonasal mucosa. These findings indicate that clinical improvement during treatment was accompanied by changes in eosinophilic inflammation and selected structural features of tissue remodeling.

## 1. Introduction

Chronic rhinosinusitis with nasal polyps (CRSwNP) is a chronic inflammatory disease of the upper airways characterized by epithelial barrier dysfunction and, in a substantial proportion of patients, type 2 inflammation [1,2]. Type 2 inflammation involves a complex interplay between T helper 2 (Th2) cells, type 2 innate lymphoid cells (ILC2s), eosinophils, mast cells, and basophils. Key mediators include interleukin (IL)-4, IL-5, and IL-13, as well as epithelial-derived alarmins such as thymic stromal lymphopoietin (TSLP), IL-33, and IL-25 [3]. Ongoing type 2 inflammation can disrupt epithelial integrity, promote inflammatory-cell accumulation, and alter the extracellular matrix. Together, these changes may reshape the sinonasal mucosa, although the mechanisms driving remodeling in CRSwNP are not fully understood.

Standard treatment of CRSwNP includes intranasal corticosteroids, with short courses of systemic corticosteroids used in patients with insufficient disease control. Endoscopic sinus surgery (ESS) is considered in patients with persistent symptoms despite appropriate medical therapy. Nevertheless, recurrence of nasal polyps remains common following surgery, particularly in patients with severe type 2 inflammation [4]. Although systemic corticosteroids and surgery can provide substantial symptomatic improvement, their effects are often temporary and do not necessarily provide sustained control of the underlying inflammatory pathways responsible for recurrent disease. Consequently, biologic therapies have become an important treatment option for patients with severe, uncontrolled CRSwNP.

According to EPOS 2020 criteria, biologic treatment may be considered in patients with bilateral nasal polyps after previous ESS who meet at least three predefined criteria, including evidence of type 2 inflammation, the need for systemic corticosteroids, significantly impaired disease-specific quality of life, loss of smell, and comorbid asthma requiring regular inhaled corticosteroid therapy [2]. Available biologic therapies act at different points of the type 2 inflammatory cascade. Dupilumab inhibits signaling through IL-4Rα, the shared receptor component for IL-4 and IL-13 [5]. Mepolizumab targets IL-5, while omalizumab neutralizes circulating IgE [6,7]. Tezepelumab, directed against TSLP, has further expanded therapeutic options for CRSwNP [8].

Despite the well-established clinical efficacy of dupilumab in CRSwNP, its effects on the histopathological features of sinonasal inflammation and tissue remodeling remain less well characterized. In particular, limited real-world data are available on longitudinal changes in tissue eosinophilia, basement membrane thickness, subepithelial edema, and Charcot–Leyden crystals (CLCs) during treatment. We therefore examined paired clinical outcomes and sinonasal histopathological findings at baseline and after 12 months of dupilumab therapy in patients with severe CRSwNP.

## 2. Materials and Methods

We conducted a prospective pilot real-world study in patients with severe CRSwNP receiving dupilumab. Eligibility required at least two previous ESS procedures, two or more oral corticosteroid (OCS) courses in the previous 12 months, and evidence of type 2 inflammation, defined as blood eosinophils >150 cells/µL, tissue eosinophilia >10 eosinophils per high-power field (HPF), or total IgE > 100 IU/mL. These criteria reflected the national eligibility requirements for biologic treatment based on EPOS 2020 and EUFOREA recommendations. A total of 27 patients were included in the study. Patients were recruited over a 6-month period. Dupilumab was administered at a dose of 300 mg every 2 weeks. Concomitant maintenance treatment, including intranasal corticosteroids and asthma controller therapy, remained unchanged throughout the 12-month observation period. In all patients, the last course of systemic corticosteroids had been completed more than 6 months before dupilumab initiation. No formal a priori sample size calculation was performed; the sample size was determined by the number of eligible patients with paired baseline and 12-month histopathological assessments available during the study period. The study was conducted in accordance with the Declaration of Helsinki and approved by the Bioethics Committee of Jan Kochanowski University in Kielce, Poland (Resolution No. 3/2025, 15 January 2025).

All patients provided informed consent for endoscopically guided sinonasal mucosal biopsy before dupilumab initiation and after 12 months of treatment. The baseline biopsy was performed within 4 weeks before the first dose of dupilumab, whereas the follow-up biopsy was obtained after at least 12 months of regular treatment. Biopsies were performed under local anesthesia with 2% lidocaine using a Pentax Medical VNL9-CP videoendoscope (PENTAX Medical, HOYA Corporation, Tokyo, Japan). Tissue samples were collected from a standardized anatomical region of the lateral nasal wall beneath the middle turbinate by the same otorhinolaryngologist at both time points.

Biopsy material was analyzed at the Department of Oncological Pathology, Holy Cross Cancer Centre, Kielce, Poland. Specimens were fixed in 10% formalin and paraffin-embedded before sectioning. H&E staining was used for routine histopathological assessment, with additional trichrome staining for basement membrane measurements. The evaluated features were basement membrane thickness, eosinophil and neutrophil counts, subepithelial edema, ulceration, and CLCs. Subepithelial edema was graded on a four-point ordinal scale (0–3), whereas ulceration was assessed as present or absent. Subepithelial edema was graded as 0 (absent: no interstitial edema and normal lamina propria architecture), 1 (mild: slightly loose edematous connective tissue with limited vascular congestion or dilation), 2 (moderate: pronounced interstitial edema with multiple dilated or congested capillaries and largely preserved architecture), or 3 (severe: marked diffuse edema involving the lamina propria, potentially extending into the submucosa, with tissue disorganization and extensive vascular dilation). CLCs were quantified by counting the number of crystals identified in the examined tissue sections and were additionally categorized as detectable or non-detectable for the primary paired analysis. Eosinophils and neutrophils were manually quantified in 10 subepithelial high-power fields (HPFs; 0.34 mm^2^ per field) using an Olympus AX60 microscope (Olympus Corporation, Tokyo, Japan) with a 40× objective and 10× ocular magnification. Mean cell counts were calculated across the examined fields and expressed as cells/mm^2^; degranulated cells were not included. Basement membrane thickness was measured manually after trichrome staining in 10 fields per specimen using CellSens software (Olympus Corporation, Tokyo, Japan). The maximum thickness measured across the 10 examined fields was retained as the patient-level value for statistical analysis. All specimens were coded and independently evaluated by two pathologists. The pathologists were blinded to the treatment time point. Discrepancies were resolved by joint review using a multi-station microscope, and a consensus final assessment was recorded.

Clinical assessments were performed at baseline and after 12 months of dupilumab treatment. Nasal polyp burden was assessed using the Nasal Polyp Score (NPS; range 0–8). Disease-specific symptoms and quality of life were evaluated using the validated 22-item Sino-Nasal Outcome Test (SNOT-22). Olfactory function was assessed using the 12-item Sniffin’ Sticks identification test. Peripheral blood eosinophil counts were also recorded at both time points.

### Statistical Analysis

Distributional assumptions were evaluated using the Shapiro–Wilk test. Approximately normally distributed variables are reported as mean ± standard deviation (SD), non-normally distributed variables as median [interquartile range (IQR)], and categorical variables as *n* (%). Age and body mass index (BMI) were approximately normally distributed, whereas the number of previous ESS procedures was not and is therefore summarized as median [IQR] [9].

For paired analyses, test selection was based on the distribution of within-patient change scores, evaluated using the Shapiro–Wilk test and Q–Q plots. Approximately normal differences were tested with a paired *t*-test; otherwise, the Wilcoxon signed-rank test was used. Binary paired outcomes were compared using the exact McNemar test, and absolute prevalence changes were reported in percentage points with 95% confidence intervals obtained from 10,000 patient-level bootstrap resamples. Because quantitative CLC counts showed marked zero inflation, CLC presence/absence was used as the primary endpoint, while quantitative CLC counts were summarized as median [IQR] and analyzed using the Wilcoxon signed-rank test. Change was defined as the 12-month value minus baseline. For outcomes analyzed using the Wilcoxon signed-rank test, paired changes were reported as Hodges–Lehmann estimates with 95% confidence intervals; for paired *t*-tests, mean differences with 95% confidence intervals were reported. For basement membrane thickness, the patient-level value used in the paired analysis was the maximum measurement obtained across the 10 examined fields at each time point. Holm correction for multiple comparisons was applied to the six histopathological endpoints and to the three main clinical endpoints: NPS, SNOT-22, and Sniffin’ Sticks. Peripheral blood eosinophil count was considered an exploratory endpoint and was therefore not included in the multiplicity adjustment. Exploratory associations between changes in histopathological parameters and changes in clinical outcomes and blood eosinophil counts were assessed using Spearman rank correlation. A *p*-value < 0.05 was considered statistically significant. No missing data were imputed; analyses were based on available paired observations.

## 3. Results

A total of 27 patients were included in the study, including 18 men (66.7%) and 9 women (33.3%). The mean age of the study population was 52.9 ± 14.0 years, with a range of 26–79 years. The mean BMI was 27.64 ± 3.40 kg/m^2^. Obesity, defined as BMI > 30 kg/m^2^, was present in 6 patients (22.2%). All 27 enrolled patients completed the 12-month follow-up, with no discontinuations or non-evaluable paired biopsies. No patient required systemic corticosteroid treatment or sinonasal surgery during follow-up. According to the EPOS response criteria, all 27 patients achieved an excellent response after 12 months of treatment. Asthma was present in 21 patients (77.8%), while NSAID-exacerbated respiratory disease (NERD) was diagnosed in 5 patients (18.5%). The median number of previous ESS procedures was 2 [IQR 2–3.5], with a range of 2–7 procedures. Baseline demographic and clinical characteristics are summarized in Table 1.

**Figure 1:**
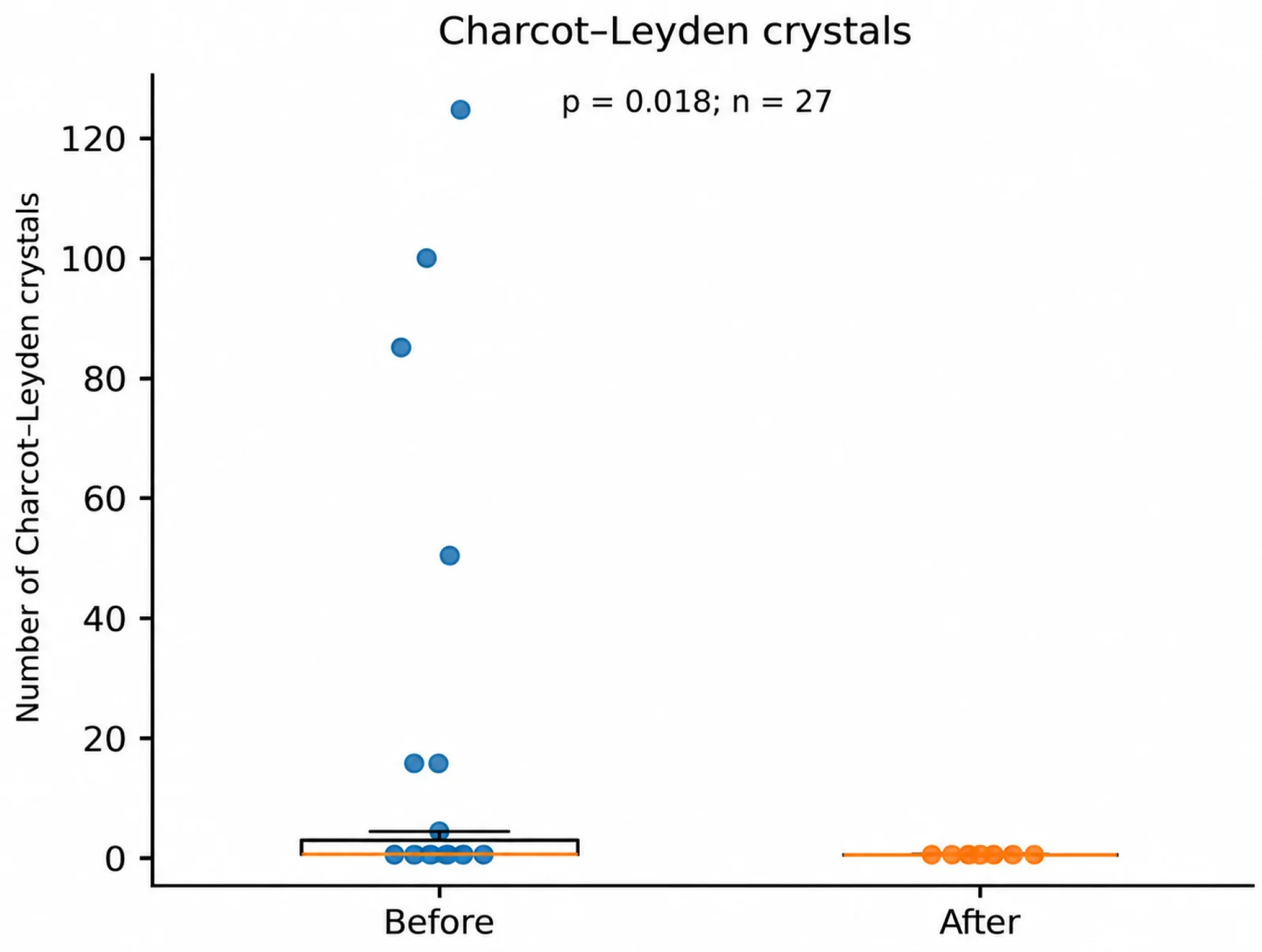
Charcot–Leyden crystal counts in H&E-stained sinonasal mucosal specimens before and after dupilumab treatment. Blue represents baseline values and orange represents post-treatment values.

**Figure 2:**
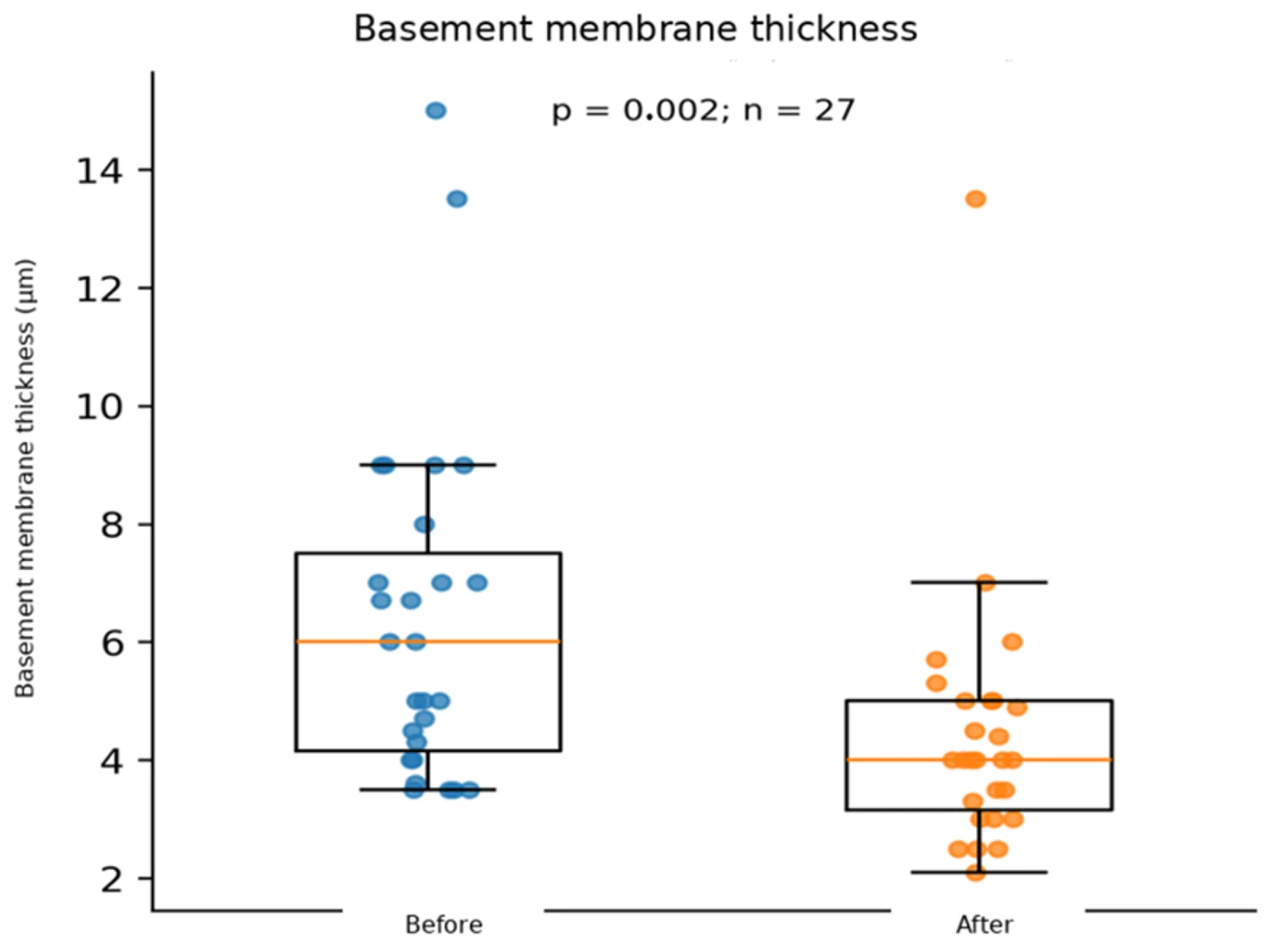
Basement membrane thickness before and after dupilumab treatment. Blue represents baseline values and orange represents post-treatment values.

**Figure 3:**
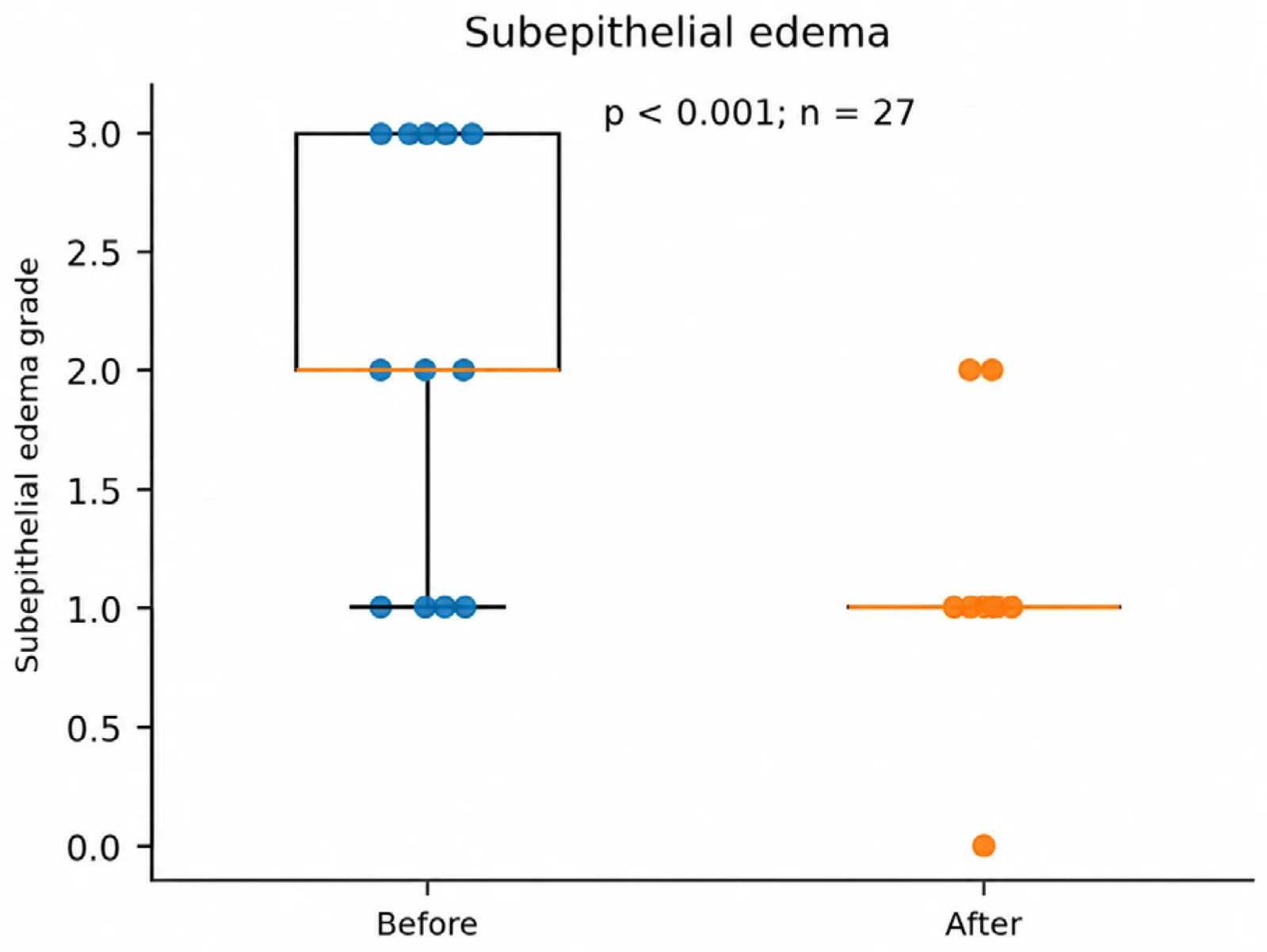
Subepithelial edema before and after dupilumab treatment. Edema severity was graded on a four-point scale as absent, mild, moderate, or severe. Blue represents baseline values and orange represents post-treatment values.

After treatment, significant reductions were observed in basement membrane thickness, tissue eosinophil count, and the severity of subepithelial edema (Table 2). All of these changes remained statistically significant after Holm correction for multiple comparisons. The most pronounced change was observed in tissue eosinophilia, with the median eosinophil count decreasing from 122 to 36 cells/mm^2^. CLC prevalence decreased from 7/27 patients (25.9%) at baseline to 0/27 after 12 months (*p* = 0.0156; Holm-adjusted *p* = 0.0469). Quantitative CLC counts and basement membrane thickness are shown in Figure 1 and Figure 2, respectively. At the individual-patient level, subepithelial edema improved in 23/27 patients (85.2%), remained unchanged in 3/27 (11.1%), and worsened in 1/27 (3.7%) (Figure 3).

Tissue neutrophil counts remained statistically unchanged. The prevalence of ulceration decreased from 74.1% to 59.3%; however, this difference did not reach statistical significance.

Clinical outcomes and peripheral blood eosinophil counts before and after 12 months of dupilumab treatment are summarized in Table 3.

After 12 months of dupilumab treatment, significant improvements were observed in all three clinical outcomes. Paired NPS and SNOT-22 data were available for all 27 patients, while Sniffin’ Sticks data were available for 26 patients. Median NPS decreased from 6.0 [IQR 5.5–7.0] to 1.0 [1.0–1.0], with a median change of −5.0 points (95% CI: −6.0 to −5.0; *p* < 0.001). The mean SNOT-22 score decreased from 73.1 ± 15.7 to 14.9 ± 10.3 points, corresponding to a mean change of −58.2 points (95% CI: −65.5 to −51.0; *p* < 0.001). Olfactory function assessed using the 12-item Sniffin’ Sticks test improved from 2.85 ± 2.46 to 7.54 ± 3.19 points, with a mean change of +4.69 points (95% CI: +3.31 to +6.07; *p* < 0.001). All three clinical outcomes remained statistically significant after Holm correction for multiple comparisons.

**Figure 4:**
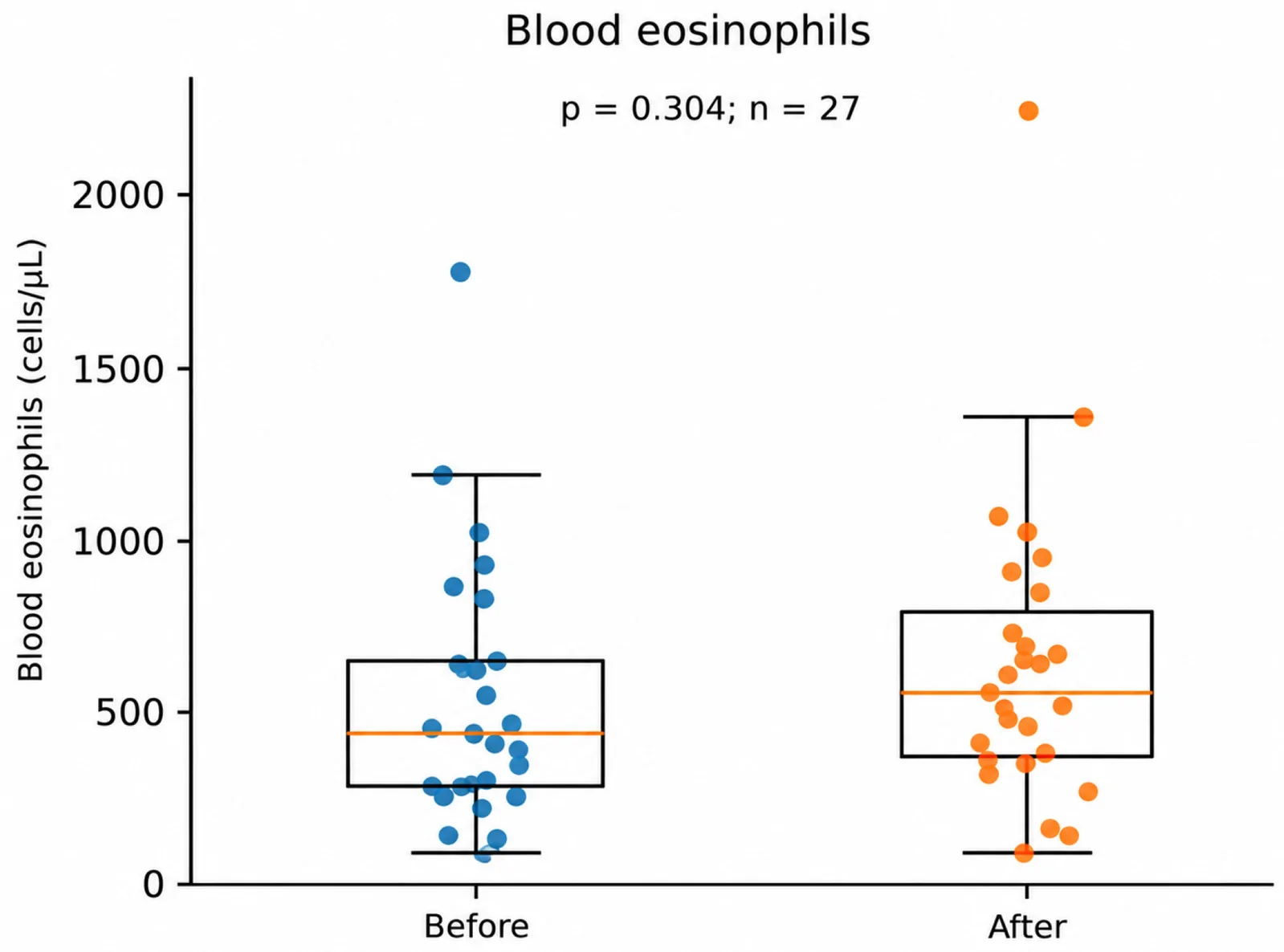
Peripheral blood eosinophil counts before and after dupilumab treatment. Blue represents baseline values and orange represents post-treatment values.

**Figure 5:**
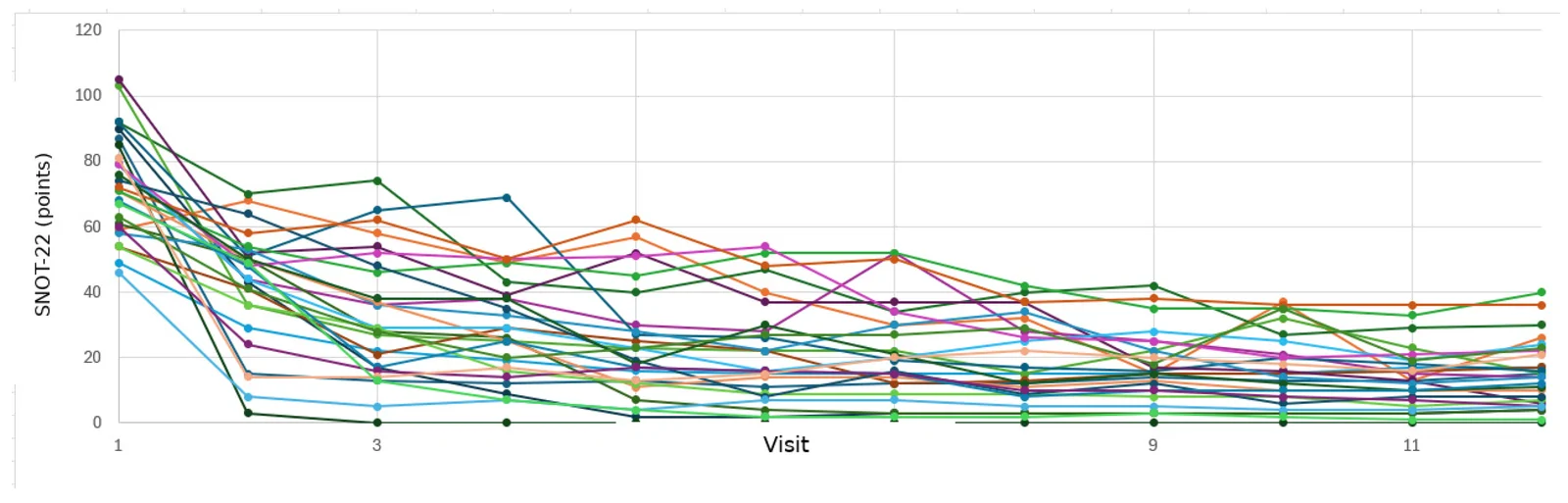
Individual trajectories of SNOT-22 scores across visits 1–12 during dupilumab treatment; visit 12 corresponds to the 12-month follow-up. Each colored line represents an individual patient.

**Figure 6:**
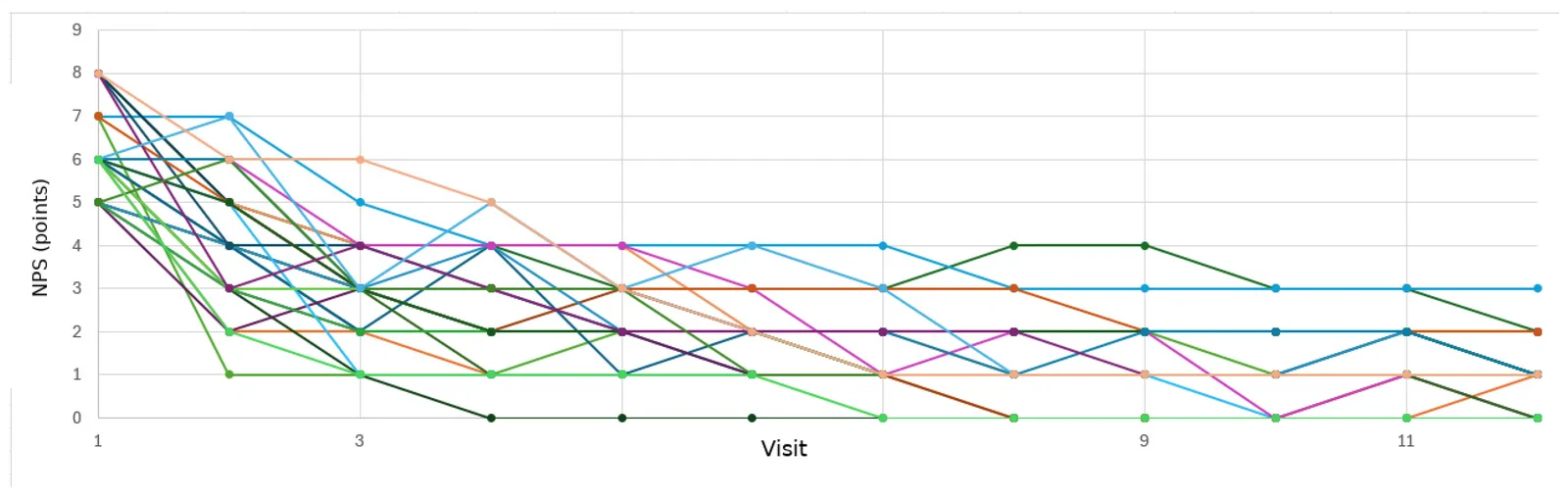
Individual trajectories of Nasal Polyp Score (NPS) across visits 1–12 during dupilumab treatment; visit 12 corresponds to the 12-month follow-up. Each colored line represents an individual patient.

**Figure 7:**
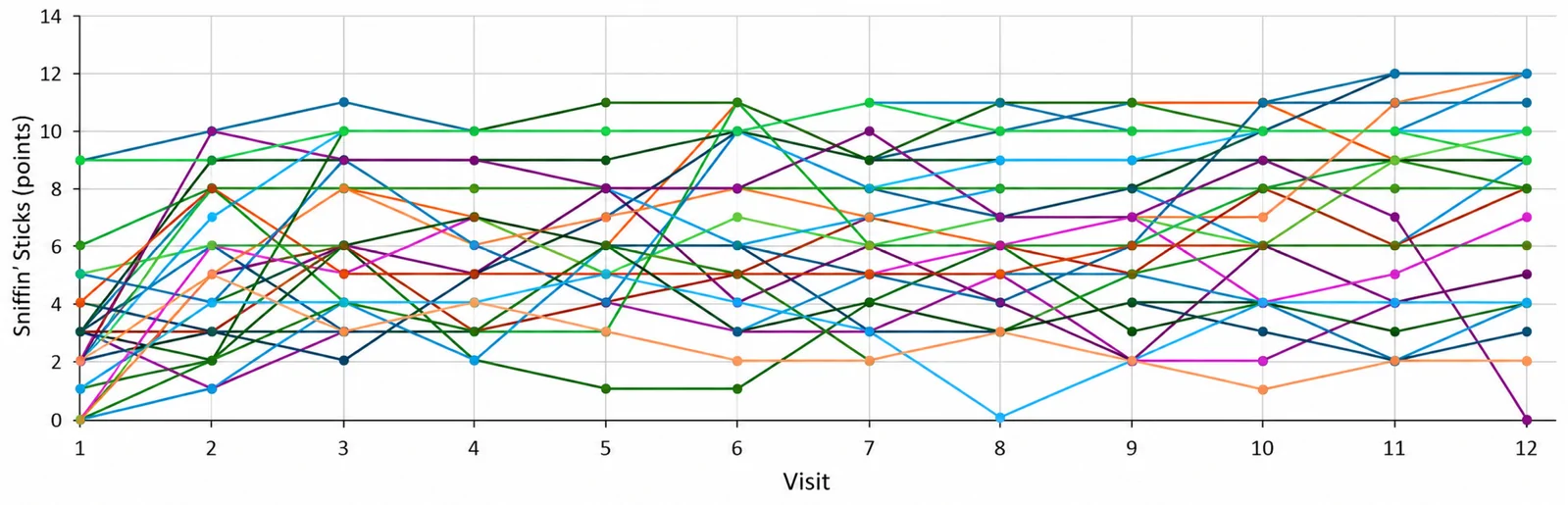
Longitudinal 12-item Sniffin’ Sticks scores across visits 1–12; the final visit represents the 12-month assessment. Each colored line represents an individual patient.

Peripheral blood eosinophil counts did not change significantly during treatment, increasing from a median of 425 [270–634] to 545 [360–780] cells/µL (median change +10 cells/µL; 95% CI: −80 to +260; *p* = 0.304) (Figure 4). Individual trajectories of SNOT-22, NPS, and Sniffin’ Sticks scores are shown in Figure 5, Figure 6 and Figure 7, respectively. 

Exploratory analyses showed no statistically significant correlations between changes in tissue eosinophil counts, basement membrane thickness, or subepithelial edema and changes in NPS, SNOT-22, olfactory function, or blood eosinophil counts (all *p* ≥ 0.122).

Representative histopathological specimens illustrating tissue eosinophilia, subepithelial edema, and Charcot–Leyden crystals are shown in Figure 8, Figure 9 and Figure 10. Additional high-resolution histopathological images are provided in Figures S1–S5.

## 4. Discussion

In this real-world cohort of patients with severe CRSwNP, the 12-month clinical response was accompanied by clear histopathological changes. Nasal polyp burden and SNOT-22 scores decreased markedly, while performance on the 12-item Sniffin’ Sticks test improved. In paired tissue samples, eosinophilic infiltration, basement membrane thickness, and subepithelial edema were all lower after treatment. CLCs detectable at baseline were no longer identified by light microscopy after therapy in affected specimens.

By targeting IL-4Rα, dupilumab interrupts signaling from both IL-4 and IL-13. Although these cytokines are not major drivers of eosinophil maturation in the bone marrow, they contribute to epithelial activation, eotaxin production, adhesion-molecule expression, and eosinophil recruitment into tissues. Their inhibition could therefore limit eosinophil trafficking to the sinonasal mucosa and reduce local type 2 inflammation. Lower eosinophil activation may also decrease eosinophil extracellular trap formation and extracellular galectin-10 release, thereby limiting CLC formation [10,11].

This interpretation is supported by molecular studies of patients with surgically refractory CRSwNP treated with dupilumab. Reduced local expression of the CLC gene, which encodes galectin-10, has been reported in sinonasal tissue following dupilumab treatment [12]. This molecular finding is consistent with a treatment-associated reduction in local eosinophilic inflammatory activity. Dorismond et al. further demonstrated that changes in CLC expression following dupilumab initiation may coexist with alterations in arachidonic acid metabolism and eicosanoid-related pathways [13]. Such molecular changes may contribute to reductions in tissue inflammation, edema, and remodeling.

Previous morphological and ultrastructural studies have documented epithelial alterations in CRSwNP and investigated tissue IL-33 expression [14]. However, relatively few studies have evaluated the effects of dupilumab at the cellular or ultrastructural level in CRSwNP. In a pilot study of 10 patients, Giombi et al. demonstrated structural improvement of the sinonasal mucosa during dupilumab treatment and suggested that patients with a more pronounced type 2 inflammatory profile may exhibit greater mucosal recovery [15]. De Corso et al. showed that local nasal eosinophilia was associated with greater disease severity [16], while Dorismond et al. reported that increased tissue eosinophilia was associated with a more favorable response to dupilumab [17]. Together with our findings, these observations support the concept that tissue-level changes may accompany and potentially contribute to the clinical response to biologic treatment.

These observations may also be considered within the concept of the “united airway” [18]. Similar histopathological effects have been reported in the lower airways. Domvri et al. performed bronchoscopic tissue sampling in patients with severe eosinophilic asthma before and after 12 months of mepolizumab treatment and demonstrated improvement in both clinical outcomes and histopathological features of airway remodeling [19]. Although the biologic targets differ, these findings suggest that inhibition of distinct pathways involved in type 2 inflammation may result in parallel clinical and structural improvements throughout the respiratory tract.

The main limitations of this study are the absence of a control group, the single-center design, the small sample size, potential regression to the mean, and potential variability in repeated biopsy sampling sites. Therefore, the observed histopathological changes should be interpreted as treatment-associated rather than directly attributable to dupilumab.

Overall, our findings show that marked clinical improvement during dupilumab therapy in severe CRSwNP was accompanied by substantial changes in the inflammatory and structural characteristics of the sinonasal mucosa. The pronounced reduction in tissue eosinophilia, together with decreased basement membrane thickness, subepithelial edema, and CLCs, indicates treatment-associated changes in eosinophilic inflammation and selected features of tissue remodeling.

## 5. Conclusions

After 12 months of dupilumab therapy, patients with severe CRSwNP had lower NPS and SNOT-22 scores and better olfactory performance. Paired tissue analysis also showed reduced eosinophilic infiltration, lower basement membrane thickness, and less subepithelial edema. Thus, clinical response occurred in parallel with changes in eosinophilic inflammation and selected features of tissue remodeling. Controlled studies are needed to determine whether these tissue changes are causally related to dupilumab.

## Figures and Tables

**Figure 8:**
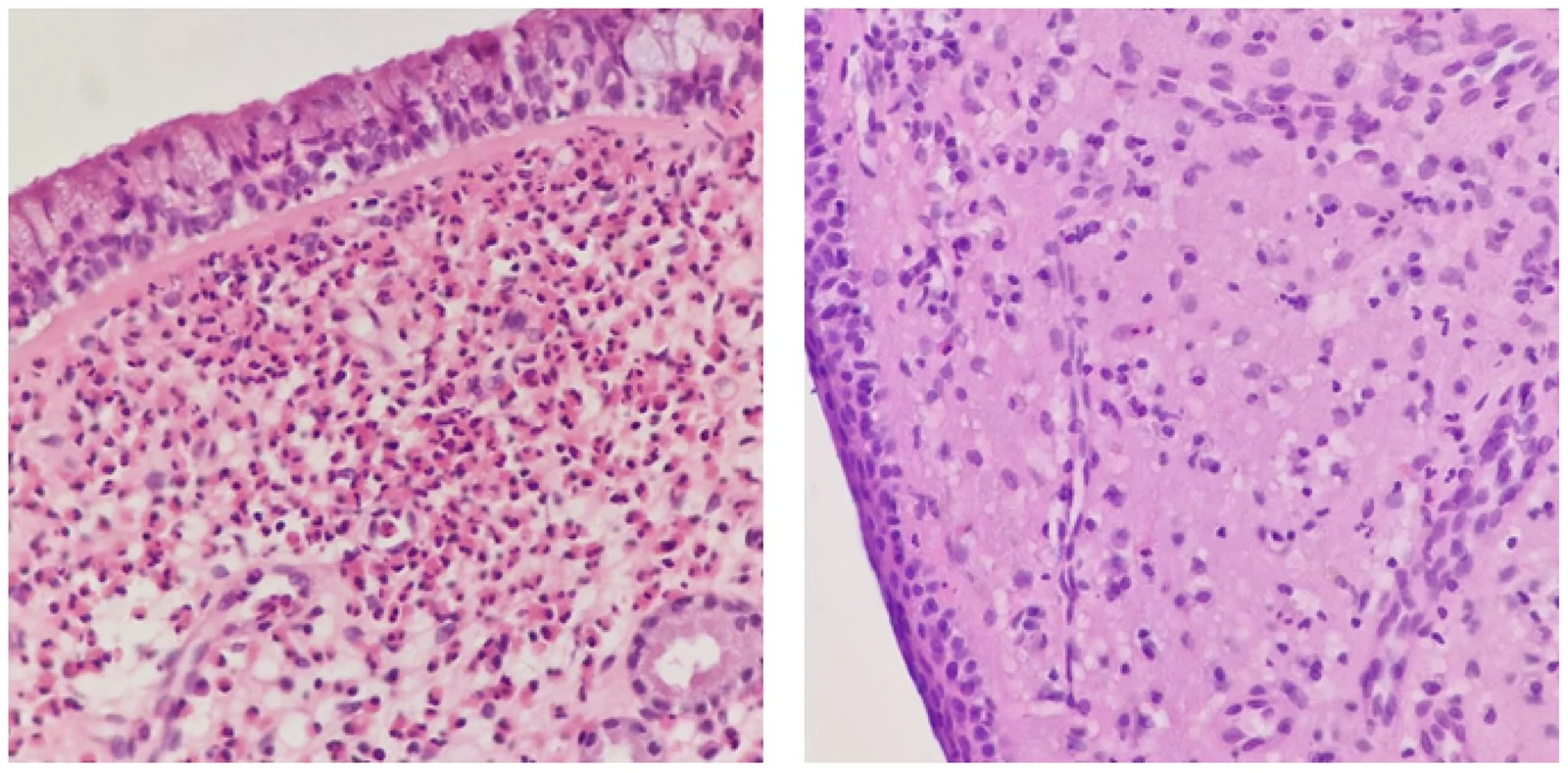
Representative H&E-stained histopathological specimens showing a reduction in basement membrane thickness and tissue eosinophilia after 12 months of dupilumab treatment.

**Figure 9:**
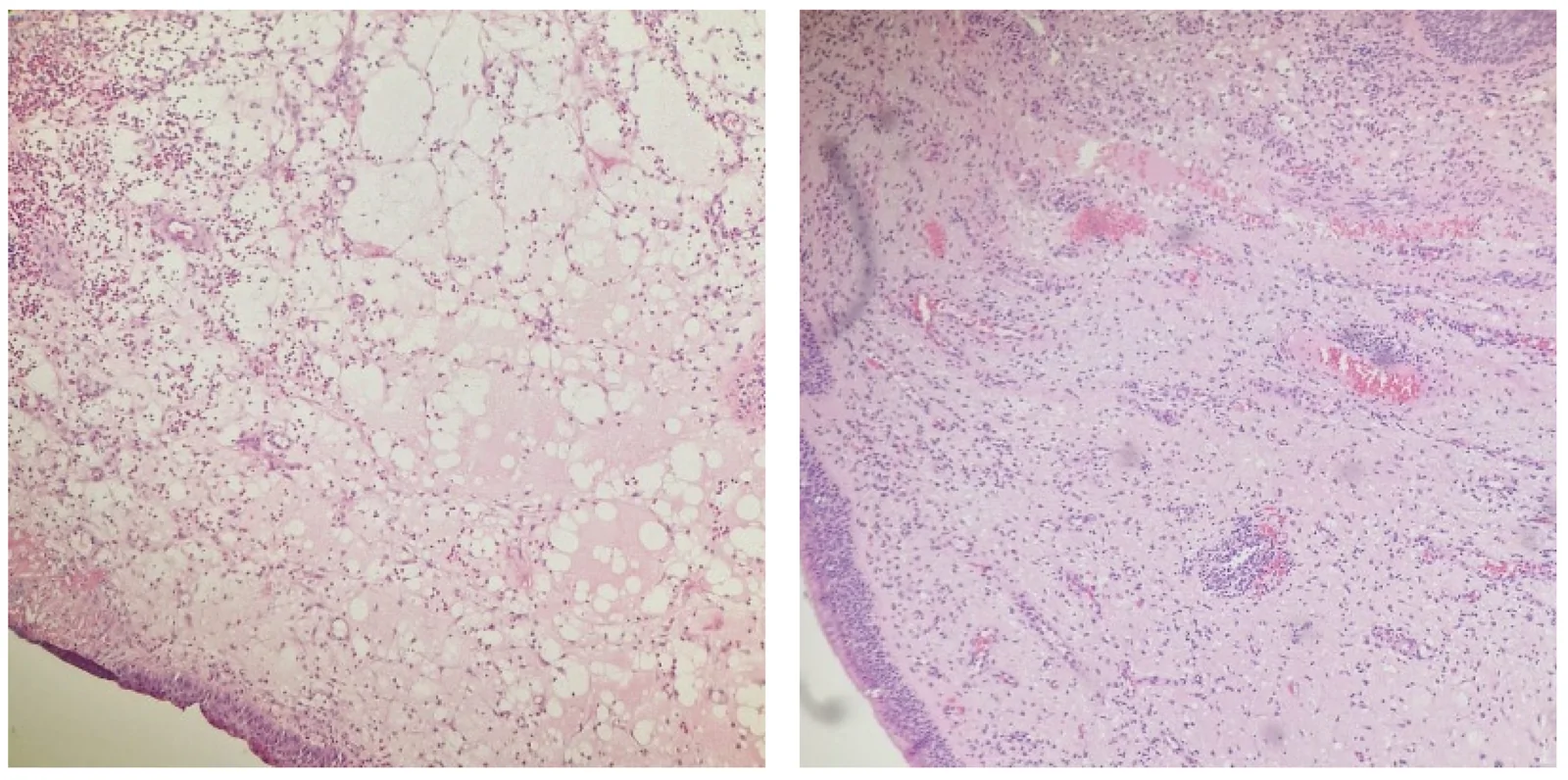
Representative H&E-stained histopathological specimens showing a reduction in subepithelial edema from moderate to mild after 12 months of dupilumab treatment.

**Figure 10:**
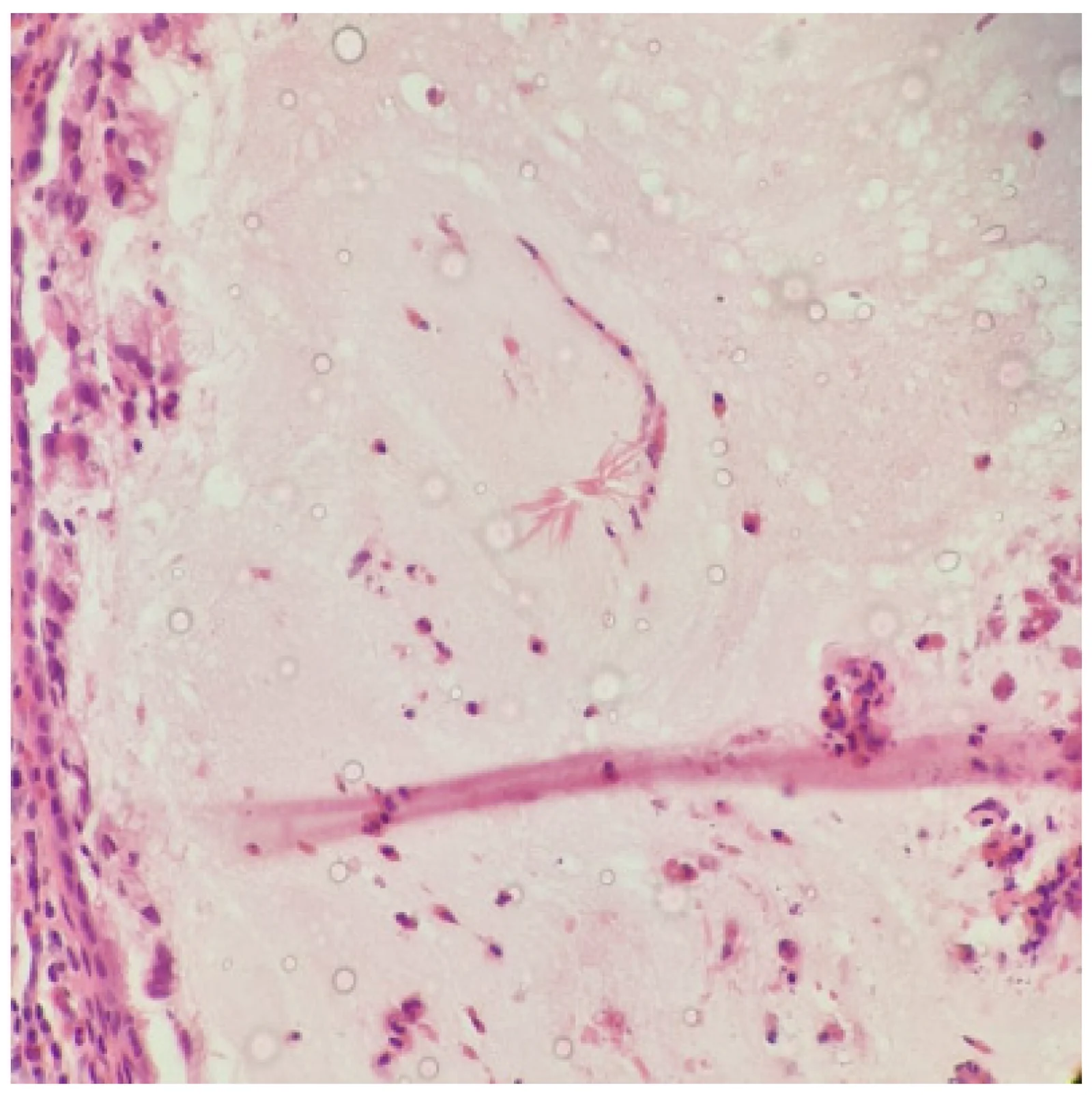
Representative H&E-stained histopathological specimen showing Charcot–Leyden crystals before dupilumab treatment.

**Table 1:** Baseline demographic and clinical characteristics of the study population.

Characteristic	Study Population (*n* = 27)
Age, years	52.9 ± 14.0
Age, median [IQR], years	51.0 [41.5–63.0]
Age range, years	26–79
Female sex, *n* (%)	9 (33.3)
Male sex, *n* (%)	18 (66.7)
BMI, kg/m^2^	27.64 ± 3.40
Obesity (BMI >30 kg/m^2^), *n* (%)	6 (22.2)
Asthma, *n* (%)	21 (77.8)
NERD, *n* (%)	5 (18.5)
Number of previous ESS procedures, median [IQR]	2 [2–3.5]
Number of previous ESS procedures, range	2–7

**Table 2:** Changes in histopathological parameters after 12 months of dupilumab treatment.

Parameter	Before Treatment	After Treatment	Change (95% CI)	*p*-Value	Holm-Adjusted *p*
Basement membrane thickness, µm	6.0 [4.15–7.5]	4.0 [3.15–5.0]	−1.75 (−2.80 to −0.75)	0.0019	0.0077
Tissue eosinophils, cells/mm^2^	122 [81–323]	36 [19.5–76]	−129.25 (−181.5 to −64.0)	<0.001	<0.001
Subepithelial edema, grade	2 [2–3]	1 [1–1]	−1.0 (−1.5 to −1.0)	<0.001	<0.001
Tissue neutrophils, cells/mm^2^	7 [4–15.5]	9 [2–12]	0.0 (−9.0 to 10.0)	0.9808	0.9808
Ulceration, *n* (%)	20/27 (74.1%)	16/27 (59.3%)	−14.8 pp (−33.3 to 0.0)	0.21808	0.4375
CLC presence, *n* (%)	7/27 (25.9%)	0/27 (0%)	−25.9 pp (−44.4 to −11.1)	0.0156	0.0469

Data are presented as median [IQR] or *n* (%), as appropriate. Change represents post-treatment minus baseline. CI, confidence interval; pp, percentage points. Continuous paired comparisons were performed using the Wilcoxon signed-rank test; ulceration and CLC presence were analyzed using the exact McNemar test. Holm-adjusted *p*-values account for multiplicity across the six histopathological endpoints.

**Table 3:** Changes in clinical outcomes and blood eosinophil count after dupilumab treatment.

Parameter	Before Treatment	After Treatment	Change (95% CI)	*p*-Value	Holm-Adjusted *p*
NPS, points (*n* = 27)	6.0 [5.5–7.0]	1.0 [1.0–1.0]	−5.0 (−6.0 to −5.0)	<0.001	<0.001
SNOT-22, points (*n* = 27)	73.1 ± 15.7	14.9 ± 10.3	−58.2 (−65.5 to −51.0)	<0.001	<0.001
Sniffin’ Sticks, points (*n* = 26)	2.85 ± 2.46	7.54 ± 3.19	+4.69 (+3.31 to +6.07)	<0.001	<0.001
Blood eosinophils, cells/µL (*n* = 27)	425 [270–634]	545 [360–780]	+10 (−80 to +260)	0.304	—

Data are presented as mean ± SD or median [IQR], as appropriate. Change represents post-treatment minus baseline. CI, confidence interval; SD, standard deviation. Holm correction was applied to NPS, SNOT-22, and Sniffin’ Sticks. Peripheral blood eosinophils were treated as an exploratory outcome; the unadjusted *p*-value is reported.

## Data Availability

The original contributions presented in this study are included in the article. Further inquiries can be directed to the corresponding authors.

## Supplementary Materials

The following supporting information can be downloaded at: https://www.mdpi.com/article/10.3390/medsci14060686/s1. Figure S1: Basement membrane before treatment; Figure S2: Basement membrane after treatment; Figure S3: Subepithelial edema before treatment; Figure S4: Subepithelial edema after treatment; Figure S5: Charcot-Leyden crystals.
